# Analysis of the influence of occupational, sociodemographic and health factors on the demotivation of the intensivist

**DOI:** 10.47626/1679-4435-2022-976

**Published:** 2024-08-05

**Authors:** Elamara Marama de Araujo Vieira, Jonhatan Magno Norte da Silva, Wilza Karla dos Santos Leite, Raynara Samille Guerra Oliveira, Luiz Bueno da Silva

**Affiliations:** 1 Departamento de Fisioterapia, Universidade Federal da Paraíba (UFPB), João Pessoa, PB, Brazil; 2 Departamento de Engenharia de Produção, Universidade Federal de Alagoas, Belmiro Gouveia, AL, Brazil; 3 Departamento de Fisioterapia, Universidade Federal do Amapá, Macapá, AP, Brazil; 4 Departamento de Engenharia de Produção, UFPB, João Pessoa, PB, Brazil

**Keywords:** occupational health, intensive care units, health personnel, saúde do trabalhador, unidade de terapia intensiva, pessoal de saúde

## Abstract

**Introduction:**

Understanding motivation, identifying motivational factors of health professionals, and recognizing how managers and leaders can successfully motivate healthcare professionals is a growing concern.

**Objectives:**

To assess the occupational, sociodemographic, and health factors that influence the occurrence of demotivation in the intensive care unit professionals.

**Methods:**

We performed a cross-sectional study with health professionals from nine intensive care units in João Pessoa, Paraíba state, Brazil. Data were collected using an adapted version of the Health Care Establishment Questionnaire. We built a Logistic Regression model to analyze the influence of variables on the motivational state, and variables were selected by the Backward method. We used 80% of the sample for parameter estimation and the remaining 20% for testing and validation. We used the R software for the analyses, with a significance level of α ≤ 0.05.

**Results:**

We identify that the variable with the greatest power over the intensivist’s demotivation was shift work (odds ratio [OR] = 4.215, p = 0.006). The number of symptoms (OR = 1.206, p = 0.000) and working time (OR = 1.080, p = 0.031) were also significant risk variables. When the three variables were combined, the professional’s chance of feeling unmotivated increased by 38 times (OR = 38.99, p = 0.000).

**Conclusions:**

Based on these results, it is possible to identify aspects that will require organizational adjustments so that intensivists remain satisfied and motivated.

## Abstract (pt)

**Introdução:**

Há uma crescente preocupação em relação a entender os fenômenos da motivação, identificar os fatores motivadores dos profissionais de saúde e reconhecer como o gestor e os líderes conseguem motivar a equipe com sucesso.

**Objetivo:**

Avaliar os fatores ocupacionais, sociodemográficos e de saúde que influenciam na ocorrência de desmotivação no profissional de saúde intensivista.

**Métodos:**

Tratou-se de um estudo transversal, realizado com profissionais de saúde de nove unidades de terapia intensiva localizadas em João Pessoa, no estado da Paraíba, Brasil. Os dados foram coletados por meio de uma versão adaptada do Health Care Establishment Questionnaire. Para análise da influência das variáveis sobre o estado motivacional, construiu-se um modelo de regressão logística, com seleção de variáveis pelo método backward. Utilizou-se 80% do total da amostra para estimação dos parâmetros, e os 20% restantes foram usados para o teste e a validação dos resultados. As análises foram realizadas no *software* R, com nível de significância de α ≤ 0,05.

**Resultados:**

Identificou-se que a variável com maior poder sobre a desmotivação do intensivista foi o trabalho em turnos (razão de chances [OR] = 4,215, p = 0,006). O quantitativo de queixas sintomatológicas (OR = 1,206, p = 0,000) e o tempo de trabalho (OR = 1,080, p = 0,031) também foram variáveis significativas sobre o risco. Quando as três variáveis estiveram combinadas, aumentou-se em 38 vezes a chance de o profissional se sentir desmotivado (OR = 38,99, p = 0,000).

**Conclusões:**

Com base nesses resultados, é possível identificar aspectos do trabalho que exigem ajustes organizacionais para que os intensivistas mantenham-se satisfeitos e motivados.

## INTRODUCTION

Employee motivation, although often neglected, is a critical challenge for health services in many countries, being one of the most difficult assets to assess and ensure, and a major determinant of performance in health services, especially in developing countries.^1,2^

Motivation is the willingness of employees to exert high levels of effort towards organizational goals, conditioned by the satisfaction of some individual needs.^3^ The level of motivation that an individual or team exerts on their task affects behavior and organizational performance, including the quality of services provided.^1^ Therefore, the overall success of the organizational project depends on the commitment of the team to the project, which is directly related to their level of motivation. In health care settings, where employees are the main resources, demotivation critically determines the success of organizations.^4^

Thus, motivation of health personnel includes determinants that drive the performance of tasks, regardless of resources and knowledge available. Motivation is a process that results from the dynamic interactions between individuals, the workplace, and the community. Failure to consider motivation among health personnel can lead to underuse of available resources and poor performance of the health system.^1^

A variety of theories set out to understand motivation and explain why people at work behave in certain ways in terms of effort. Porter & Lawler^5^ postulated one of the most widely accepted theories: they proposed a model of work motivation based on intrinsic and extrinsic factors. Intrinsic motivation refers to doing something for the inherent satisfaction involved and is autonomous, while extrinsic motivation means doing something to obtain tangible rewards. This model suggested that intrinsic and extrinsic rewards are additive and represent total job satisfaction.

Some studies have explored particular aspects of motivation, such as Ferraro et al.,^6^ who analyzed the relationship between motivation, length of service, and workload in Portuguese and Brazilian populations; Heyns & Kerr,^3^ who investigated the differences between generations; Štefko et al.,^7^ who focused on gender differences; and Snelgar et al.,^8^ who analyzed cultural and sociodemographic aspects. However, most of these studies were conducted in developed countries with a different social fabric and occupational situation from developing countries, such as Brazil, which makes comparisons questionable, as the factors related to intrinsic and extrinsic motivation vary between these contexts.

Challenges faced by the health industry, including technological advances, the metamorphosis occurring in the demographics and diversity of the workforce, restructuring, current events faced by professionals, such as the crisis in health systems, especially driven by the COVID-19 pandemic, the degradation and impoverishment of professional practice, and the constant changes in the health demands of populations, making it more essential than ever to understand employees needs in order to promote high-quality health services and create a healthy workplace.^9^

Therefore, given the complexity of the biodynamic environment in which health personnel are placed, it is extremely important that they remain adherent to the organizational objectives and at full physical and mental capacity. However, these professionals are exposed to countless adversities, arising from the precariousness of their work and the risks inherent in the environment in which they work.

Thus, it is a matter of growing concern to understand the phenomena of motivation, to identify the motivating factors of health personnel, and to recognize how managers and leaders can successfully motivate their teams.^10^ Given that sociodemographic and organizational factors can explain around 30% of the variations in the motivation scores of healthcare personnel^11^ and that all employees in an organization have a roughly similar set of needs, which allows organizations to predict the characteristics that should be present at work,^2^ we wonder which variables could most strongly influence the motivational state of this group of professionals.

This study assessed occupational, sociodemographic, and health factors that influence demotivation among intensive care health personnel. As a result, an occupational profile of these workers can be drawn up, and efforts can be optimized to implement management measures that the organization should focus on to improve their health and productivity. The result is expected to be an improvement in employees’ health, an improvement in the organization production process and, in terms of health services, an improvement in the care provided to patients.

## METHODS

### STUDY DESIGN AND FIELD OF STUDY

This is an observational, cross-sectional study. The study was ethically approved and conducted in 9 intensive care units (ICUs) for adults in the public, municipal and state-run health network in the city of João Pessoa, PB, Brazil, corresponding to 100% of the facilities in the city. These included 3 ICUs specifically for trauma patients, 2 for obstetric patients, and the remainder for the general public.

### SAMPLE AND RECRUITMENT

This study used a no probabilistic consensual sample. The final sample consisted of all eligible professionals working in the wards investigated who agreed to participate in the study. The study sample included health personnel working in the selected units of analysis, in full professional practice, of both sexes, and no age restriction. Physicians, nurses, physical therapists, and nursing assistants who agreed to answer the questionnaire were considered eligible.

### DATA COLLECTION

Health personnel were informed about the scope of the study and upon their agreement were asked to sign an informed consent form. For data collection, an adaptation with selected questions from the Health Care Establishment questionnaire proposed by the Department of Occupational and Environmental Medicine^12^ was used, which contained questions to identify the interviewee and their health and wellbeing conditions. Functional status and self-perceived symptoms were assessed using a 37-psychological and physical signs and symptoms checklist reported over the previous 15 days, allowing the interviewee to include other signs and symptoms. Similarly, the questionnaire included the interviewee’s work experience, working hours, and lifestyle.

This questionnaire was completed during the interviewee’s working hours, with prior instructions provided by the interviewer. A single interviewer was given priority for the entire sample, so that there would be no duplicates during the instructions for filling in the questionnaires. The confidentiality of the interviewee’s identity was emphasized throughout the entire data collection process.

### VARIABLES

The self-reported state of demotivation was considered the dependent variable. This study assigned a value of 0 to indicate “not demotivated” and 1 to indicate “demotivated”. The independent variables included sex (0 = male), occupation (1 = physician; 2 = nurse; 3 = nursing assistant; 4 = physical therapist), age (continuous variable), weight (continuous variable), height (continuous variable), weekly workload (up to 45 hours per week = 0; > 45 hours per week = 1), number of ICUs in which the individual works (discrete variable), work shifts (up to 2 shifts = 0; 3 shifts = 1), length of service (continuous variable), medical appointments in the last quarter (no = 0; yes = 1), exercise (0 = no), continued use of medication (no = 0; yes = 1), previous diagnoses of illnesses (0 = no), perception of the ICU environment (1 = good; 0 = fair; 2 = bad) and the total number of signs and symptoms that the individual complains about out of the 37 investigated (discrete variable). In the models described below, these signs and symptoms are not treated individually, but in relation to how many each individual in the sample identified as present.

### STATISTICAL ANALYSIS

Initially, the data collected in the field was analyzed descriptively, considering measures of central tendency and frequency of occurrence of discrete and categorical variables. As the data was collected directly by an interviewer, no data was lost. At this stage, the internal consistency of the questionnaire was assessed using Cronbach’s alpha.

The modeling was built using the statistical technique of logistic regression using the backward variable selection method and parameters of 5% significance for the variable to remain and 10% for it to leave the model. The backward elimination technique initially includes all the dependent variables in the model and sequentially removes those that do not make a significant contribution. It is a process of trial and error to find the best solution to the problem;^13^ however, it should be noted that even before they were included in the selection process, the theoretical implications of these dependent variables in relation to the outcome were observed.

We used 80% of the total sample to estimate the parameters, and the remainder 20% was used for testing and validation. The level of fit of the logistic model was assessed using pseudo R^2^. To validate the model, we used the graphical performance criterion obtained through the area under the curve of the receiver operating characteristic (AUC ROC), which suggests predictions of reliability and error rate for the results obtained using the logistic regression model. According to Hosmer & Lemeshow,^13^ the accuracy of the model is classified as acceptable when the AUC ROC values are between 0.7 and 0.8.

When we identified which variables among those investigated actually contribute significantly to the motivational state of the intensivist specialist, we investigated the interaction of these variables, the odds ratios (e^**β**^) and the respective p values resulting from each situational framework. All the tests were completed using R with a significance level of α ≤ 0.05.

## RESULTS

Data collection instrument had a Cronbach’s alpha of 0.76 (95% confidence interval 0.71-0.81), which was considered sufficient to proceed with the analysis. A total of 128 health personnel were interviewed, and their sociodemographic, occupational, and health characteristics are shown in Table 1.

**Table 1:** Sociodemographic, occupational, and health characteristics of the sample

	Mean	SD	%
Age	35.5	8.2	
BMI	26.8	5.6	
Length of service	7.4	5.9	
Women			80.5
Occupation			
Physician			7.8
Nurse			18.0
Physical therapist			20.3
Nursing assistant			53.9
Weekly workload up to 45 hours Number of ICUs in which the individual works			53.1
1			60.9
2			32.8
3			3.9
4			1.6
5			0.8
Perception of the ICU environment where they work			
Good			25.7
Fair			57.9
Bad			16.4
Works 3 shifts			65.6
Medical appointments in the last quarter			36.7
Exercise			35.9
Previous diagnoses of illnesses			22.6
Continued use of medication			56.2
Feels demotivated			42.2

SD = standard deviation; BMI = body mass index; ICU = intensive care unit.

Table 1 shows a sample of health personnel who are predominantly experienced women in their practice. However, they have poor occupational characteristics, given that a considerable number work more than 45 hours per week, in more than 1 ICU, and on duty both day and night shift.

Table 1 also shows that these health personnel are young, yet poorly exercise, take medication continuously and, given the length of time, attend medical appointments frequently. Table 2 shows the percentages of all 37 signs and symptoms investigated. The health personnel had an overall mean of 9 symptomatic complaints (standard deviation = 7.3), and the most prevalent were psychological complaints such as boredom, mood swings, anxiety, and stress. The most common physical symptoms were headaches, sore throats, muscle pain, physical fatigue, and muscle tension.

**Table 2:** Perception of the 37 signs and symptoms investigated

Psychological symptoms	%		%		%
Boredom	73.4	Irritability	48.4	Lethargy	8.6
Mood swings	60.2	Difficulties concentrating	32.8	Nervousness	29.7
Memory changes	35.2	Stress	62.5	Loss of appetite	11.7
Anxiety	59.3	Insomnia	35.9	Anger	37.5
Depression	8.6				
Physical symptoms					
Tinnitus	30.5	Sneezing	50.0	Watery eyes	16.4
Itching, burning or irritation of the eyes	37.5	Fatigue	60.9	Palpitation	27.3
Headache	64.8	Visual fatigue	30.5	Hearing loss	8.6
Irritated, stuffy or runny nose	47.6	Pharyngitis	32.8	Rhinitis	32.8
Sore throat	55.5	Hoarse, dry throat	32.8	Dry skin	29.7
Muscle pain	66.4	Hypertension	20.3	Tachycardia	21.1
Muscle tension	53.9	Skin irritation	16.4	Mucosal irritation	17.2
Dizziness	20.3	Eye redness	17.9	Cough	31.3

The statistical model obtained through logistic regression involved the intercept and 3 explanatory variables, modeled based on the values of the β estimates shown in Table 3. As a result of the backward variable selection technique, Table 3 only shows the model parameters that were significant in all the modeling phases, given that in each phase of removing nonsignificant variables (p > 0.05), the model parameters change, which generates a substantial number of parameters for each variable.

**Table 3:** Parameters of the model

	β	Odds ratio (e^β^)	p-value^*^
Intercept	-4.534	0.010	
Work shift	1.438	4.215	0.006
Length of service	0.077	1.080	0.031
Number of symptoms	0.187	1.206	0.000

* Likelihood-ratio test.

Table 3 shows that all variables have positive coefficients, indicating that when these categorical variables take on higher values, the likelihood of the individual feeling demotivated is also higher.

Based on the odds ratios (e^β^) and their corresponding p-values shown in Table 3, it is clear that the variable that has the greatest influence on the demotivation of intensivists is their work shifts (odds ratio = 4.215, p < 0.05). Thus, working multiple shifts, night and day, increases the worker’s risk of feeling demotivated 4-fold. Every year, professionals are 8% more likely to feel demotivated in relation to their work, and with each additional symptom, they are 20% more likely to feel demotivated. Considering the large number of nosological occurrences reported (Table 2), with a mean of 9 symptoms per employee, the risk becomes more representative for the population investigated.

In the likelihood-ratio test (binomial model), showing the occurrence of demotivation as a response (Table 3), the factors investigated were statistically significant in relation to the response variable. The pseudo R^2^ of the model was 0.472 and the AUC ROC was 0.830, indicating that it is adjusted and suitable for assessing the risk of worker demotivation, and may even be suitable for classification.

The odds ratios shown in Table 3 indicate the isolated influence of each independent variable on the intensivist motivational state. Considering that these variables can occur simultaneously in the intensivist, their interactions need to be analyzed in order to produce the response variable. The interaction between the dependent variables (Table 4) indicates that, together, these parameters are significant in the model in question (p < 0.05).

**Table 4:** Odds ratios of the interaction between the variables in the model

Work shifts	Length of service (years)	Number of symptoms	β	Odds ratio (eβ)	p-value^*^
<3	<7.4	<14		Baseline	
>3	<7.4	<14	0.167	1.181	0.822
<3	>7.4	<14	-0.479	0.619	0.700
<3	<7.4	>14	0.996	2.708	0.245
>3	>7.4	<14	0.878	2.407	0.300
>3	<7.4	>14	1.648	5.200	0.032
<3	>7.4	>14	1.754	5.777	0.078
>3	>7.4	>14	3.663	38.99	0.000

Bold = significant values.

The interaction analysis between the variables indicates that individuals who work 3 shifts, who have been working for over 7.4 years, and who report having 14 or more health symptoms have a 38-fold higher risk of feeling demotivated (p = 0.000). If the employee has worked for less than 7.4 years, they still have a 5-fold higher risk of feeling demotivated (p = 0.032) compared to other employees.

## DISCUSSION

The results previously presented showed that the variables relative to the number of work shifts, length of service, and number of symptoms are significantly relevant to the employee self-report of demotivation. However, work shift was the variable with the greatest weight among them. The impact of work shifts on motivation is added to all the deleterious effects on health that this organizational arrangement can have. Work shifts are associated with a higher prevalence of negative work-related factors and inadequate habits and lifestyle, such as weight gain and changes in blood pressure and sleep, impacting on the employee general health, vitality, and functional capacity.^14,15^

Working multiple shifts in a row stems from the need to make ends meet and unfavorable organizational adjustments, causing damage to social life due to interference in personal relationships and family life, difficulties in planning routines,^16^ and confinement, given that the ICU is a restricted access ward.

The participation of employees in the organization of their working hours allows for an improvement in their working conditions, considering individual chronobiological characteristics when scheduling shifts. In critical moments, an efficient strategy for adjusting demands would be to use additional teams to reduce the need for exhausting sequences of work shifts.^17^ Therefore, the employee chronobiological rhythm with the work shift can be a factor of quality of life and well-being.^18^

When work tasks provide employees with a sense of self-development and self-efficacy, they feel more satisfied, and satisfied employees have higher levels of general perception of motivation.^19,20^ Motivated health professionals have better perceived results, both in terms of their general state of health and their work performance, with less burnout and fewer physical symptoms.^21^

Therefore, the strongest drivers of all dimensions of motivation are nonfinancial managerial tools, so policymakers and health workforce stakeholders should focus on these tools to remedy motivation problems.^10,22^ Intrinsic and sociocultural factors are important motivators, such as respect, career growth, the relationship between work and family and time for social life.

Extrinsic nonfinancial incentives and human resource management tools play an important role in increasing the motivation of health personnel. These include improved leadership and support, better use of performance evaluation for decisionmaking, creation of development opportunities, work schedule adjustments, provision of necessary materials and communication that strengthens relationships of trust between workers, supervisors, managers, the organization and patients.^4,23,24^ Demotivating factors in all settings are mainly organizational, such as fewer qualification opportunities, less personal security, and poor working conditions.^25,26^

Thus, considering the importance of health care services, the demotivation of health care personnel can have major consequences for the performance of their work and for the process of treating and healing patients. Poor work structures, high workloads, and unfavorable salaries that lead to double shifts favor demotivation, becoming a strong factor in the risk of human errors in health care, which can delay or even harm the patient clinical condition.^9,10,27^

### STRENGTHS AND LIMITATIONS

Some issues that limit the explanatory power of the results should be noted, but at the same time pave the way for future research. These include a more objective assessment of work characteristics rather than self-reporting. The evaluation of additional health parameters (e.g. sick leave) in a longitudinal design would facilitate causal inferences.

### CONTRIBUTIONS AND RECOMMENDATIONS

The criteria of length of service and frequency of health complaints, also cited as important predictors of demotivation, can be used to screen out workers at greater risk of demotivation, for whom it may be necessary to make organizational adjustments to keep them satisfied and motivated at work. These results can contribute to targeting intervention measures aimed at optimizing the quality of work of intensive care health personnel, and can also be used to identify measures that can be implemented to improve the fit between employee and work, improving working conditions and reducing the burden of illness imposed by unhealthy conditions.

## CONCLUSIONS

Working multiple shifts was the variable that most increased intensivists risk of demotivation. When intensivists work multiple shifts, their risk of becoming demotivated is 4-fold higher compared to other employees. The number of symptoms intensivists experience can be a strong indicator of their motivational state, given that with each additional symptom, they are 20% more likely to feel demotivated, which may suggest that the general health of employees affects their motivation at work.
